# Adenoid cystic carcinoma of the breast: Experience at a tertiary care centre of Northern India

**DOI:** 10.1016/j.ijscr.2018.08.035

**Published:** 2018-08-31

**Authors:** Namita Bhutani, Pradeep Kajal, Sham Singla

**Affiliations:** a Deptt. of Pathology, PGIMS, Rohtak, Haryana, India; b Deptt. of Pediatric Surgery, PGIMS, 23/8FM, Medical campus, Rohtak, Haryana, India; c Deptt. of Surgery, SGT Medical College, Budheda, Gurgaon, Haryana, India

**Keywords:** Adenoid cystic carcinoma, Breast neoplasms, Immunohistochemistry, Triple-negative breast carcinoma

## Abstract

**Introduction:**

Adenoid cystic carcinoma of the breast (breast-ACC) is a rare tumor with a favorable prognosis, despite its triple-negative status and special type of basal-like tumor for which scant population-based descriptive data exist. We sought to provide new population-based information on breast-ACC in India. Due to the paucity of the number of cases, the natural history of the disease is not fully understood. This study was undertaken to examine the clinico-pathological characteristics of the disease and to evaluate the outcome of surgical intervention in a tertiary referral care centre.

**Materials and methods:**

A retrospective analysis of all patients diagnosed and treated for ACC Breast in our hospital over the past 10 years was carried out (2005–2015). A database of the characteristics of these patients was developed. In all, 14 patients were identified. The investigations performed included routine blood investigations, chest X-ray, bone scan and either an ultrasound or a CT scan.

**Results:**

During the time period of 10 years, of 2347 with breast malignancy admitted to our department, only 14 were diagnosed as having ACC (3.15%). All patients were women (100%). The patients had a median age of 60.7 years (range 37–81). The most common symptom was lump in the breast. Two patients (14.2%) presented with nipple and skin retraction and two patients (14.2%) were asymptomatic with the diagnosis made by an incidental finding on routine examination. The CT and/or magnetic resonance imaging (MRI) showed the typical features of carcinoma breast. All the 14 patients were taken up for surgery. Nine patients underwent Modified radical mastectomy and five patients underwent Breast conservation surgery. Axillary lymph node dissection was carried out in seven patients and sentinel lymph node biopsy in the remaining. Tumor cells had a characteristic histologic pattern of ACC of the breast. Perineural invasion was present in six cases.

**Discussion:**

ACC of the breast is a very rare malignancy, accounting for less than 0.1% of all breast neoplasms. It affects the left and right breasts equally and tumors arise irrespective of the breast quadrants. However, in about 50 percent of patients, lesions are found in subareolar region. Pain or tenderness described in the minority of cases has not been correlated with histologically-confirmed perineural invasion. ACC is categorized as a basal-like subtype of breast carcinoma. Most cases are macroscopically well-circumscribed. Occasionally, pink, tan, or gray microcysts are evident. A tumor typically consists of a dual-cell population of luminal and myoepithelial-basal cells which may be arranged in one or more of three architectural patterns: tubular-trabecular, cribriform, and solid-basaloid. There is no consensus on the optimal management for patients with ACC of the breast. Based on its indolent clinical course and favorable outcome, ACC of the breast is generally cured by breast-conserving surgery, such as wide excision or quadrantectomy with or without radiotherapy.

**Conclusion:**

Breast-ACC among women is characterized by ER-negative/PR-negative expression, rare regional lymph node involvement, a favorable prognosis with excellent survival, and absence of associated cancers. These findings reinforce the importance of tailored treatments for breast-ACC and the apparent heterogeneity of basal-like breast cancers.

## Highlights

• Adenoid cystic carcinoma (ACC) of the breast is a rare type of primary breast cancer accounting for less than 0.1% of all primary breast cancers.
• Unlike other triple negative breast cancers that are associated with poor prognosis, ACC has an overall excellent prognosis.
• Mammographically, these tumors may appear as asymmetric densities or irregular masses. Sonographically, they appear as well-defined, irregular, heterogeneous, or hypoechoic masses. Nonetheless, the radiographic findings are non-specific and can be misdiagnosed as benign lesions. Subsequently, it could be challenging for a radiologist to make the correct diagnosis of carcinoma without histologic confirmation.
• Due to the rarity of the tumor and large variations in the patterns of practice, guidelines for treatment have not been established. Because of these distinct clinicopathologic features that set it apart from the other triple negative breast cancers, an understanding of ACC of the breast is essential for surgical pathologists, breast surgeons, and oncologists.

## 1 Introduction

Adenoid cystic carcinoma (ACC) of the breast is a rare type of primary breast cancer accounting for less than 0.1% of all primary breast cancers [1]. ACC of the breast, previously called “cylindroma”, was first described by Geschickter and Copeland in 1945 [2]. The majority of ACCs arising in the breast have been localized disease with a low rate of axillary lymph node involvement and distant metastases. A characteristic histologic pattern of ACC of the breast includes both epithelial and myoepithelial components and resembles a well-known tumor of the salivary gland origin known by the same name. The tumor comprises luminal epithelial cells and myoepithelial-like cells with tubular, cribriform, or solid architectural patterns. ACC of the breast belongs to the basal like subgroup of breast cancers [[3], [4], [5]]. Based on extensive molecular and genetic profiling studies, basal-like tumors are most often hormone receptor [estrogen receptor (ER) and progesterone receptor (PR)] negative, do not express human epidermal growth factor receptor 2 (Her2), but express one or more basal/myoepithelial cell markers [*e.g*., cytokeratins (CKs) 5, 5/6, 14 and 17] [6]. C-kit overexpression as well as MYB overexpression from frequent t(6;9) (q22-23;p23-24) translocation, which fuses the MYB oncogene to transcription factor NFIB, have been reported [[7], [8], [9]]. Unlike other triple negative breast cancers that are associated with poor prognosis, ACC has an overall excellent prognosis [10]. The 10-year survival rate for patients with ACCs of the breast has ranged from 85% to 100% [11,12]. Due to the rarity of the tumor and large variations in the patterns of practice, guidelines for treatment have not been established. Because of these distinct clinicopathologic features that set it apart from the other triple negative breast cancers, an understanding of ACC of the breast is essential for surgical pathologists, breast surgeons, and oncologists. This aim of this report is to present our experience with patients with ACC of the breast and to review the clinicopathologic features and current preferred treatment modalities.

## 2 Materials and methods

A retrospective analysis of all patients diagnosed and treated for ACC in our hospital over the past 10 years was carried out (2005–2015). A database of the characteristics of these patients was developed, including age, gender, tumor location (data were derived from radiological investigations or surgical records) and size (data were derived from radiological investigations or surgical records and finally confirmed by pathology), treatment (data were derived from the medical records, including the types of surgery), and histopathological and immunohistochemical features. In all, 14 patients were identified. A Computed Tomography scan (CT Scan) was performed in all the patients and the findings revealed a mass in the breast. Pre-operative fine needle aspiration cytology (FNAC) was performed in 5/14 patients. All the patients who underwent resection were followed up every 6 months. The investigations performed included routine blood investigations, chest X-ray, bone scan and either an ultrasound or a CT Scan of the chest.

## 3 Results

**Fig. 1:**
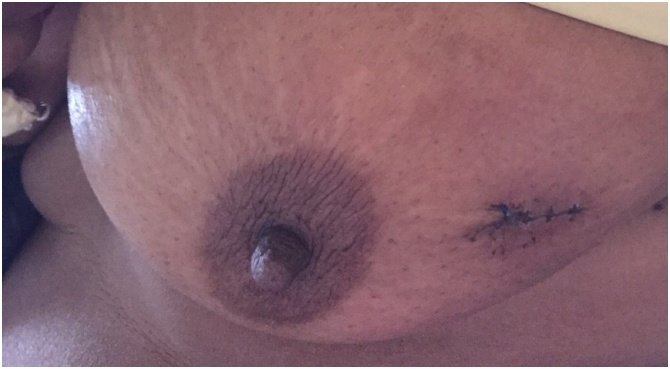
Clinical picture demonstrating lump in the breast (right side).

**Fig. 2:**
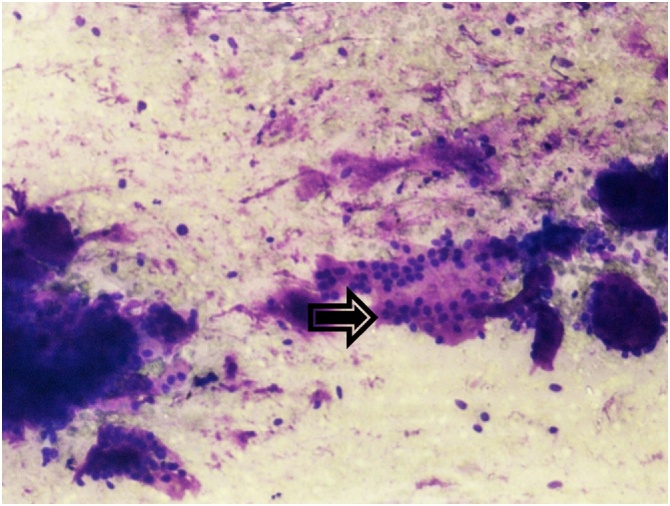
FNAC smears revealing clusters of basaloid cells (arrow) with hyaline globules (Giemsa 200×).

**Fig. 3:**
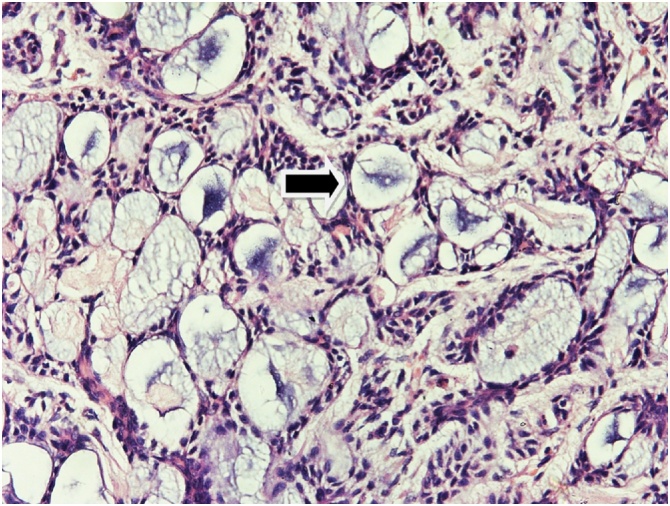
Hematoxylin and eosin staining demonstrate nests and islands of tumor cells with sharply outlined luminal spaces, which are filled with mucinous material (arrow) (cylindrical type) [200×].

**Fig. 4:**
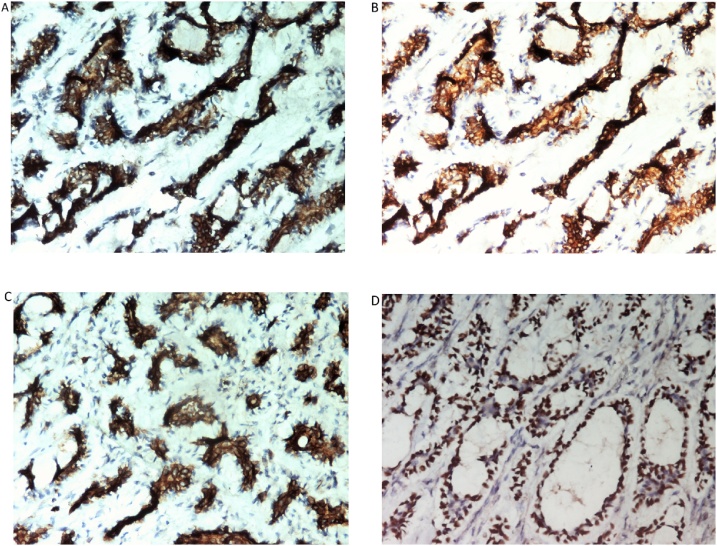
A: Tumor cells are positive for cytokeratin (IHC; 200×). B: Tumor cells are positive for epdermal growth factor receptor. (IHC; 200×). C: Tumor cells are positive for C-KIT. (IHC; 200×). D: Tumor cells are positive for MYC. (IHC; 200×).

During the time period of 10 years, 2347 patients with breast malignancy admitted to our department, only 14 were diagnosed as having ACC (3.15%). All patients were women (100%). The patients had a median age of 60.7 years (range 37–81). The most common symptom was lump in the breast (71.4%). Two patients (14.2%) presented with nipple and skin retraction and two patients (14.2%) were asymptomatic with the diagnosis made by an incidental finding on routine examination. The CT and/or magnetic resonance imaging (MRI) showed the typical features of carcinoma breast. In 8 patients the growth was in left breast (57.1%) while in rest were in right breast (Fig. 1). In two patients the tumor was located in the central region (14.2%), in four patients in the upper outer quadrant (28.5%), in three in the upper inner (21.4%), three in lower outer quadrant (21.4%) and in the remaining two patients in the lower inner quadrant (14.2%). Nine patients were postmenopausal (64.2%) and rest were premenopausal (35.7%). Pre-operative fine needle aspiration cytology (FNAC) was performed in 5/14 patients (Fig. 2). In 4 patients, features were suggestive of adenoid cystic carcinoma, while in one it was inadequate. All the 14 patients were taken up for surgery. Nine patients underwent Modified radical mastectomy and five patients underwent Breast conservation surgery. Axillary lymph node dissection was carried out in seven patients and sentinel lymph node biopsy in the remaining. Patient characteristics are summarized in Table 1, Table 2. Local reccurence was seen in two patients, while distant metastasis in three cases. At histopathological examination, tumor mass in the breast was seen. The tumor comprises luminal epithelial cells and myoepithelial-like cells with tubular, cribriform, or solid architectural patterns. Tumor cells had a characteristic histologic pattern of ACC of the breast includes both epithelial and myoepithelial components and resembles a well-known tumor of the salivary gland origin known by the same name (Fig. 3). The immunohistochemistry profiles are summarized in Table 4 and shown in Fig. 4. Perineural invasion was present in six cases. Postoperative radiotherapy was given to 11 patients and adjuvant chemotherapy to 6 patients (Table 3). The average postoperative hospital stay was 5.6 days.

**Table 1:** Clinical characteristics.

S. No.	Age	Gender	Laterality	Site	Menopausal status	Clinical features
1.	75	F	Left	UOQ	Postmenopausal	Lump
2.	69	F	Right	LIQ	Postmenopausal	Skin retraction
3.	37	F	Left	UIQ	Premenopausal	Routine screening
4.	48	F	Left	Central	Premenopausal	Nipple retraction
5.	63	F	Right	LOQ	Postmenopausal	Lump
6.	58	F	Left	LOQ	Postmenopausal	Lump
7.	49	F	Right	LIQ	Premenopausal	Lump
8.	47	F	Right	UIQ	Premenopausal	Routine screening
9.	55	F	Left	UIQ	Postmenopausal	Lump
10.	52	F	Right	UOQ	Postmenopausal	Lump
11.	61	F	Left	LOQ	Postmenopausal	Lump
12.	85	F	Left	UOQ	Premenopausal	Lump
13.	81	F	Left	UOQ	Postmenopausal	Lump
14.	71	F	Right	Central	Postmenopausal	Lump

F: Female, UIQ: Upper inner quadrant, UOQ: Upper outer quadrant, LIQ: Lower inner quadrant, LOQ: Lower outer quadrant.

**Table 2:** Pathological features.

S. No.	TNM STAGING	Perineural Invasion	Local Recurrence	Distant Metastasis
1.	T2N0M1	+	No	Yes (Lung)
2.	T1N0M0	–	No	No
3.	T2N0M0	–	No	No
4.	T2N0M0	–	No	No
5.	T1N0M1	+	No	Yes (Liver)
6.	T1N0M0	–	No	No
7.	T2N0M0	+	No	No
8.	T2N0M0	+	No	No
9.	T2N0M1	+	No	Yes (Bone)
10.	T2N0M0	–	No	No
11.	T2N0M0	–	No	No
12.	T1N0M0	+	Yes	No
13.	T1N0M0	–	No	No
14.	T2N0M0	–	Yes	No

**Table 3:** Treatment outcomes.

S. No.	Surgery	Axillary Management	Post OP RT	Adjuvant CT	DFS (MO)	OS (MO)
1.	MRM	SLNB	Yes	No	30	32+
2.	MRM	ALND	No	Yes (CAP)	36	40+
3.	BCS	SLNB	Yes	No	28	38+
4.	BCS	SLNB	Yes	No	32	40+
5.	MRM	SLNB	Yes	Yes (CMF)	24	36+
6.	MRM	ALND	No	Yes (FAC)	48	75+
7.	BCS	ALND	Yes	No	45	100+
8.	BCS	ALND	Yes	No	75	48+
9.	MRM	SLNB	Yes	Yes (CMF)	24	27+
10.	MRM	SLNB	Yes	Yes (CMF)	32	45+
11.	MRM	ALND	Yes	No	64	85+
12.	BCS	ALND	No	No	35	40+
13.	MRM	SLNB	Yes	No	37	52+
14.	MRM	ALND	Yes	Yes (CAP)	21	38+

RT: Radiotherapy, CT: Chemotherapy, DFS: Disease free survival, OS: Overall survival, MRM: Modified radical mastectomy, BCS: Breast conservation surgery, ALND: Axillary lymph node dissection, SLNB: Sentinel lymph node biopsy, CAP: Doxorubicin, cyclophosphamide and cisplatin, CMF: Cyclophosphamide, methotrexate 5-fluorouracil, FAC: Fluorouracil, doxorubicin, and cyclophosphamide.

**Table 4:** Immunohistochemical characteristics.

S.NO.	ER	PR	HER 2NEU	EGFR	CK5/6	C KIT	MYB
1.	–	FOCAL +	–	+	+	–	–
2.	–	–	–	+	+	+	+
3.	–	–	–	+	+	–	–
4.	–	–	–	+	+	–	–
5.	–	–	–	+	+	–	–
6.	–	–	–	FOCAL +	+	–	–
7.	–	–	–	+	+	–	–
8.	–	–	–	+	+	–	–
9.	–	–	–	+	+	–	–
10.	–	–	–	+	+	+	+
11.	–	–	–	+	+	–	–
12.	–	–	–	+	+	–	–
13.	–	–	–	+	+	–	–
14.	–	–	–	+	+	–	–

## 4 Discussion

ACC of the breast is a very rare malignancy, accounting for less than 0.1% of all breast neoplasms [1]. Most patients with ACC have a breast mass at first presentation, but nipple discharge is unusual. A few cases have also been detected incidentally on routine screening mammograms of asymptomatic patients [13]. It affects the left and right breasts equally and tumors arise irrespective of the breast quadrants. However, in about 50 percent of patients, lesions are found in subareolar region. Pain or tenderness described in the minority of cases has not been correlated with histologically-confirmed perineural invasion [2]. Mammographically, these tumors may appear as asymmetric densities or irregular masses. Sonographically, they appear as well-defined, irregular, heterogeneous, or hypoechoic masses. Nonetheless, the radiographic findings are non-specific and can be misdiagnosed as benign lesions. Subsequently, it could be challenging for a radiologist to make the correct diagnosis of carcinoma without histologic confirmation [14]. Although most patients present with a solitary tumor, a few cases of multifocal ACC of the breast have also been reported [15].

ACC is categorized as a basal-like subtype of breast carcinoma, which is supported by microarraybased gene expression profiling studies [16]. A recent Comparitive Genomic Hybridisation (CGH) analysis study revealed that ACC of the breast manifested significantly lower frequencies of genetic instability and lower copy number alterations than the histologic grade-matched basal-like and invasive ductal carcinomas of NST. At the genomic level, ACC is substantially different from the other basal-like breast cancers. Studies show that it rarely harbors genomic aberrations associated with basal-like invasive ductal carcinomas of No Special Type (NST), such as gains of 1q, 6p, 8q, and 10p, and losses of 4p, 5q, and 10q. Furthermore, aneuploidy is reported in fewer than 10% of cases with ACC of the breast. Together, these findings illustrate the heterogeneity of triple-negative, basal-like breast cancers. In addition, the miR-24 was decreased in salivary gland-derived but overexpressed in breast-derived adenoid cystic carcinomas. Similar to ACCs of the salivary gland, ACCs of the breast are characterized by the t(6;9) (q22-23; p23-24) chromosomal translocation, which generates fusion transcripts involving the oncogene *MYB* and the transcription factor gene *NFIB*. Approximately 7% of breast cancer cases are related to hereditary conditions and caused by mutations in the *BRCA1* and *BRCA2* genes. Although medullary and metaplastic breast carcinomas, with which ACC shares immunohistochemical and molecular findings, show a frequent promotor methylation of *BRCA1* gene, ACC of the breast usually retains normal *BRCA1* gene function [10]. Finally there are several recent studies that identified potential breast ACC biomarkers. Insulin-like growth factor-mRNA-binding protein 3 (IMP3) is an oncofetal protein and a component of the insulin-like growth factor pathway. Studies indicate that it could serve as a biomarker for basal-like breast carcinomas, and a recent report showed that the IMP3 is commonly overexpressed in ACCs of the breast [17].

Most cases are macroscopically well-circumscribed. Occasionally, pink, tan, or gray microcysts are evident. A tumor typically consists of a dual-cell population of luminal and myoepithelial-basal cells which may be arranged in one or more of three architectural patterns: tubular-trabecular, cribriform, and solid-basaloid [18]. There are two types of structures lined by these two different types of cells: true glandular spaces and pseudolumina. Luminal cells, characterized by round nuclei and eosinophilic cytoplasm, surround true gland lumina containing periodic acid-Schiff-positive neutral mucin. The tumor is graded as I, II, or III based on the proportion of a solid element, which correlates with prognosis [19]. The myoepithelial-like cells lining the cribriform spaces occasionally contain basement membrane-like materials that can be myxoid or mucinous. Because cribriform architecture is the most commonly observed pattern in ACC of the breast, the major differential diagnosis for ACC includes cribriform carcinoma. Cribriform carcinoma comprises only epithelial cells and no basement membrane-like materials in the spaces and usually expresses ER and PR, whereas ACC of the breast usually does not express ER, PR, and HER2 and tends to express CK5/6 or EGFR and EGFR is potentially an important therapeutic target [10]. Although triple-negative breast cancer (TNBC) generally has high proliferative activity, several studies using proliferative markers (e.g., Ki-67) reported a low proliferation rate in ACC of the breast. Immunohistochemically, the luminal cells are positive for CK7, CK8/18, epithelial membrane antigen, and CD117 (c-Kit). The myoepithelial-basal cells are immunoreactive for basal cytokeratins (CK5, CK5/6, CK14, CK17), myoepithelial markers (p63, actin, calponin, S-100 protein), vimentin, and epidermal growth factor receptor (EGFR) [10]. ACC of the breast should be distinguished from invasive cribriform/ tubular carcinoma or a benign condition termed collagenous spherulosis [20]. This is especially important when a pathologist is provided with tiny tissue specimens obtained by core needle biopsies. In addition, limited evidence exists of c-Kit and/or p63 immunoreactivity in ACCs of the breast (positive for both), compared to the invasive cribriform/tubular carcinomas which are negative for both markers [21]. In collagenous spherulosis, collagenous spherules are irregular, mostly observed at the periphery of the lesions, and no mucosubstance is detected within lumina. Immunohistochemically, ACCs are c-Kit (+), calponin (-), and smooth muscle myosin (-), whereas collagenous spherulosis lesions are c-Kit (-), calponin (+), and smooth muscle myosin (+), which may help to differentiate between these two types of lesions [22]. The differential diagnosis of the solid (basaloid) variant of ACC includes small cell carcinoma (neuroendocrine carcinoma), solid papillary carcinoma, metaplastic carcinoma, and malignant lymphoma. C-kit, a transmembrane tyrosine kinase receptor protein encoded by the proto-oncogene KIT, is highly expressed in ACC of the salivary gland (89–100%) and breast (83–100%) [7,8]. Unlike those tumors, though, KIT mutation in ACC is very rare, and the precise mechanism for C-kit overexpression is still unknown. ACCs with C-kit overexpression without mutation show no response to treatment with tyrosine kinase inhibitor. In our series, few cases also showed strong nuclear staining for MYB, revealing its overexpression. MYB may play a key role in the molecular pathogenesis of ACC and is expected to be a candidate therapeutic target.

There is no consensus on the optimal management for patients with ACC of the breast. Based on its indolent clinical course and favorable outcome, ACC of the breast is generally cured by breast-conserving surgery, such as wide excision or quadrantectomy with or without radiotherapy [23]. Mastectomy is recommended for invasive lesions when a cosmetically satisfactory excision is not possible, especially when the tumor has a high-grade pattern [24]. Leeming et al. [25], who evaluated 24 cases, found that 37.5% of patients had local recurrence of ACC after undergoing local excision only. Although the relationship between margin status and recurrence may not be clear, local excision alone may result in unacceptably high rates of local recurrence. Due to the low rate of axillary lymph node metastasis, the role of ALND for patients with ACC remains unclear. Several retrospective studies have demonstrated that adjuvant radiotherapy may be effective for local control and survival in patients with ACC of the breast. A recent large retrospective study that included 376 patients with ACC of the breast identified by the Surveillance, Epidemiology, and End Results (SEER) database found that adjuvant radiotherapy after local surgical therapy improved both overall survival and disease-specific survival [26]. Moreover, because a high rate of positive surgical margins has been detected following breast conserving surgery, adjuvant radiotherapy may be beneficial [27]. Furthermore, while some clinicians recommend systemic adjuvant chemotherapy for patients with high-grade lesions or axillary lymph node/distant metastasis, its role in breast ACC patients remains controversial. When patients with ACC demonstrate local recurrence or distant metastases, a prolonged and indolent clinical course is still likely. However, long-term follow- up is recommended, since their long clinical course carries a risk of secondary malignancies and the risk of distant metastases increases with time [28]. As treatment of cancer enters a new stage with the development of targeted therapies, the common *MYBNFIB* fusion gene may provide new therapeutic avenues for the management of advanced ACC of the breast. Consequently, further functional studies investigating the biological consequences of the *MYB* gene of function due to the *MYB*-*NFIB* fusion are needed. Gene silencing experiments are also necessary to demonstrate that *MYB* expression is required for the survival of cancer cells with genetically activated *MYB* [29].

Distant metastases from ACC of the breast are uncommon, and they tend to occur without lymph node involvement [25]. The most frequent site of metastasis is lung; other sites include bone, liver, and kidney, which are also the metastatic sites in patients with ACC of the salivary glands [12]. Although ACCs of the breast generally have a favourable clinical course, distant metastasis and late recurrence can arise, and long term follow-up is required. Unlike other triple- negative breast cancers that are associated with poor prognosis, ACC has an overall excellent prognosis [3]. Because of these distinct clinicopathologic features that set it apart from the other triple-negative breast cancers, an understanding of ACC of the breast is essential for surgical pathologists, breast surgeons, and oncologists.

## 5 Conclusion

The correct classification of the histological special types of breast cancer is not just an academic exercise, as it has both prognostic and predictive implications. Although the majority of triple-negative, basal-like breast carcinomas are high-grade tumors, ACC is a subgroup of low grade tumors with an indolent clinical behavior that also displays a triple-negative, basal-like phenotype. Because of its low incidence, there have been only few comprehensive studies of ACC of the breast, which is one of the major limitations of this study. Awareness of the favorable clinical behaviour of breast-ACC is important, and these findings emphasize the need for clinicians to balance the risks and benefits of cytotoxic therapy given the excellent long-term survival. We also report two cases of ACC of the breast, a rare tumor known to be a basal-like subtype of breast carcinoma with overexpression of C-kit and MYB protein. Further molecular studies of these overexpressed proteins may lead to future therapeutic strategies.

## Conflicts of interest

There is no conflict of interest amongst the authors.

## Funding source

There was no source of funding for our research.

## Ethical approval

Ethical approval has been taken for the publication of this case series from the Ethics Committee, Pt BDS PGIMS Rohtak, Haryana, India.

Reference number: EC/2005/144 Dated 23.04.2005.

## Consent

Written informed consent was obtained from the patients for publication of this case series and any accompanying images.

We state that the work has been reported in line with the PROCESS criteria [30].

We also declare that there are no conflicts of interest amongst the authors.

We also state that there was no source of funding for this study.

The consent has been taken from the patients for publication of this case series.

## Author contributions

Namita Bhutani – Reviewed the literature and wrote the article.

Pradeep Kajal – Gave important inputs regarding the management of the cases and did the final editing of the article.

Sham Singla – Managed the operative and post-operative part of the patient care.

## Registration of research studies

Researchregistry3390.

## Guarantor

Pradeep Kajal.

## Provenance and peer review

Not commissioned, externally peer-reviewed.
